# High-expression of the innate-immune related gene UNC93B1 predicts inferior outcomes in acute myeloid leukemia

**DOI:** 10.3389/fgene.2023.1063227

**Published:** 2023-01-18

**Authors:** Qiaoli Li, Hong Pan, Zhen Gao, Weiwang Li, Lele Zhang, Jingyu Zhao, Liwei Fang, Yajing Chu, Weiping Yuan, Jun Shi

**Affiliations:** ^1^ Regenerative Medicine Clinic, State Key Laboratory of Experimental Hematology, National Clinical Research Center for Blood Diseases, Haihe Laboratory of Cell Ecosystem, Institute of Hematology and Blood Diseases Hospital, Chinese Academy of Medical Sciences & Peking Union Medical College, Tianjin, China; ^2^ Center for Stem Cell Medicine and Department of Stem Cell & Regenerative Medicine, State Key Laboratory of Experimental Hematology, National Clinical Research Center for Blood Diseases, Haihe Laboratory of Cell Ecosystem, Institute of Hematology and Blood Diseases Hospital, Chinese Academy of Medical Sciences and Peking Union Medical College, Tianjin, China

**Keywords:** acute myeloid leukemia, UNC93B1, prognostic and therapeutic biomarker, innate immune, metabolism, BCL2

## Abstract

Acute myeloid leukemia (AML) is a heterogeneous hematological malignancy with dismal prognosis. Identification of better biomarkers remained a priority to improve established stratification and guide therapeutic decisions. Therefore, we extracted the RNA sequence data and clinical characteristics of AML from The Cancer Genome Atlas (TCGA) and Genotype-Tissue Expression database (GTEx) to identify the key factors for prognosis. We found *UNC93B1* was highly expressed in AML patients and significantly linked to poor clinical features (*p* < 0.05). We further validated the high expression of *UNC93B1* in another independent AML cohort from GEO datasets (*p* < 0.001) and performed quantitative PCR of patient samples to confirm the overexpression of *UNC93B1* in AML (*p* < 0.005). Moreover, we discovered high level of *UNC93B1* was an independent prognostic factor for poorer outcome both in univariate analysis and multivariate regression (*p* < 0.001). Then we built a nomogram model based on *UNC93B1* expression, age, FAB subtype and cytogenetic risk, the concordance index of which for predicting overall survival was 0.729 (*p* < 0.001). Time-dependent ROC analysis for predicting survival outcome at different time points by *UNC93B1* showed the cumulative 2-year survival rate was 43.7%, and 5-year survival rate was 21.9%. The differentially expressed genes (DEGs) between two groups divided by *UNC93B1* expression level were enriched in innate immune signaling and metabolic process pathway. Protein–protein interaction (PPI) network indicated four hub genes (*S100A9*, *CCR1*, *MRC1* and *CD1C*) interacted with *UNC93B1*, three of which were also significantly linked to inferior outcome. Furthermore, we discovered high *UNC93B1* tended to be infiltrated by innate immune cells, including Macrophages, Dendritic cells, Neutrophils, Eosinophils, and NK CD56dim cells. We also found *UNC93B1* had a significantly positive correlation with *CD14*, *CD68* and almost all Toll-like receptors. Finally, we revealed negatively correlated expression of *UNC93B1* and *BCL2* in AML and conjectured that high-UNC93B1 monocytic AML is more resistant to venetoclax. And we found high *MCL-1* expression compensated for *BCL-2* loss, thus, we proposed MCL-1 inhibitor might overcome the resistance of venetoclax in AML. Altogether, our findings demonstrated the utility of *UNC93B1* as a powerful poor prognostic predictor and alternative therapeutic target.

## Introduction

Acute myeloid leukemia (AML) is a heterogenous malignancy of bone marrow which is characterized by clonal expansion and differentiation arrest in myeloid progenitor cells (De Kouchkovsky and Abdul-Hay, 2016), patients used to have rarely optional treatment, largely depending on cytarabine + anthracycline (7 + 3) intensive chemotherapy (Maganti et al., 2018), the most common treatment for AML. Although transplantation is an effectively curable therapy for AML patients, relapse is common and associated with quite poor prognosis (Christopher et al., 2018). Evidence suggested that AML relapse after transplantation was associated with dysregulated pathways that might involve in immune response, such as the process of antigen presentation (Christopher et al., 2018). Diagnosis and treatment of AML have improved over the past few decades, but overall survival (OS) remains less than 50% (Walter and Estey, 2015), and the overall 5-year survival is still low, about 24% (Shallis et al., 2019). Tumor escape in AML is expected to be reversible and an urgent problem needed to be solved. Current strategies offer alternative immunotherapeutic options, and the adaptive arm of the immune system is largely harnessed (Au et al., 2020), such as checkpoint inhibitors PD1/PD-L1 antibodies, which has been lagging far behind solid tumors for its limited efficacy in AML (Liu, 2021). Recently, accumulating evidences suggest that innate immunity might also play an important role in hematopoietic malignancies (Tettamanti et al., 2022), however, the specific mechanisms for some of these events remained unclear.

UNC-93 homolog B1 (UNC93B1), encoding an ER protein with 12 transmembrane domains (Maschalidi et al., 2017), is a key regulator of nucleotide-sensing toll-like receptors (TLRs) (Pelka et al., 2018) that sense invading pathogens and deliver them from the endoplasmic reticulum to their respective endosomal signaling (Kim et al., 2008). Toll-like receptors are the key components of the innate immune system (Sulaiman et al., 2017) and trigger a host defense response (Akira et al., 2006). UNC93B1 is associated with multiple immune diseases, such as systemic lupus erythematosus, influenza and herpes simplex encephalitis (Casrouge et al., 2006; Nakano et al., 2010; Lafferty et al., 2014). Furthermore, It has been reported that UNC93B1 is linked to human tumors such as oral squamous cell carcinomas (Wagai et al., 2019), colon cancer (Zhao et al., 2019), CML (Shokeen et al., 2018), lymphoma (Wang et al., 2016). And recent study revealed that UNC93B1 phenotype is related to survival outcomes after unrelated bone marrow transplantation (Uchino et al., 2021), however, the role of UNC93B1 in AML remains elusive.

This study is aimed at describing the clinical implications and survival-predicted functions of UNC93B1 in AML, and the potential interaction between AML blasts’ metabolism and innate immune response, considering the innate immune system such as innate immune cells and TLR signaling may overcome the current barriers in AML treatment.

## Materials and methods

### Data acquisition and processing

The RNA-seq data in transcripts per million (TPM) format and relevant clinical information from the TCGA AML cohort were obtained from the TCGA data portal (https://portal.gdc.cancer.gov/projects) (Colaprico et al., 2016). A total of 173 patients with AML and 70 normal people (The GTEx Consortium atlas of, 2020) were included in the TGCA LAML program, and Log2 transformation of the TPM RNA-seq data was performed before further intrasample comparison. 153 AML patients were eligible and extracted for survival analysis after excluding patients without clinical survival outcome.

### Quantitative real-time PCR of patient samples

Bone barrow mononuclear cells (BMNC) were Isolated from six AML patients and three healthy donors by Histopaque®-10771 (Sigma-Aldrich) standard procedure. All participants provided written informed consent. The study was approved by ethics review boards of our institution. The clinical information of enrolled patients was listed in Supplementary Table S1. Total RNA of BMNC was isolated with TRIzol reagent (Life Technologies) and then reverse transcribed to complementary DNA (cDNA) using the Evo M-MLV RT Kit (AG) with gDNA Clean. The cDNAs were then mixed with SYBR reagent, Applied Biosystems real-time PCR system were used for gene expression analysis, and the expression level were normalized to GAPDH. The primer sequences for UNC93B1 and GAPDH are shown in Supplementary Table S2.

### Establishment of risk-scoring model

Patients were divided into two groups based on the median cutoff value of *UNC93B1* expression, and the power of *UNC93B1* expression to distinguish AML from healthy individuals was estimated using the receiver operating characteristic (ROC) curve drawn by the pROC software (Schlicker et al., 2007). Various alternative clinical events were analyzed using univariate Cox analysis to evaluate their potential impact on overall survival (OS) in AML patients. Furthermore, a multivariate Cox regression model with independent prognostic factors was applied to construct the final prognostic predictors. Independent factors were then recruited to build the nomogram prognostic model (Motwani et al., 2018). Additionally, calibration (Karapanagiotis et al., 2018) was used to estimate the predictive power of the nomogram model. The nomogram and calibration curve were constructed using the “RMS” R package. Finally, the time-dependent receiver operating characteristic (ROC) analysis (Krischer et al., 2019) was performed to assess the predicted value of *UNC93B1* in the prognostic model.

### Identification of differentially expressed genes

Based on the median expression of *UNC93B1* mRNA, AML patients were divided into two groups (UNC93B1-lower and UNC93B1-higher groups). The differentially expressed genes (DEGs) between the high-UNC93B1 and low-UNC93B1 groups were identified by DESeq2R (Love et al., 2014) package. DEGs were considered here as an adjusted *p* value < 0.05 and an absolute logarithmic 2-fold change (|log2 FC|) >2.0.

### Functional pathway analysis

Functional Gene Ontology annotations were performed using R-package clusterProfiler (Yu et al., 2012), including biological process (BP), cellular component (CC), and molecular function (MF) (Gene Ontology Consortium, 2021), as well as Kyoto Encyclopedia of Genes and Genomes (KEGG) analysis (Kanehisa et al., 2022). To further evaluate the gene function, we also conducted the Gene Set Enrichment Analysis (GSEA) analysis (Szklarczyk et al., 2019) using hallmark gene sets (h.all.v7.2. symbols.gmt) from the MsigDB and it was defined as a statistically significant item with the *p* value was less than 0.05.

### Protein–protein interactions network construction and hub genes identification

Protein–protein interaction (PPI) networks can discover and annotate the biological interactions between cellular proteins. In this study, potential protein–protein interactions were predicted by the website of STRING (https://string-db.org/) (Szklarczyk et al., 2019). The PPI for up-regulated DEGs were constructed and visualized by Cytoscape (version 3.7.1) (Shannon et al., 2003). Then hub genes were found using three different algorithms. Finally, the ggplot2 package was used to map the correlation of *UNC93B1* and the expression of the hub genes in AML.

### Correlation analysis of immune infiltration and immune related genes

To explore the association between UNC93B1 expression levels and abundance of infiltrating immune cells, we used GSVA (version 1.34.0) to perform the ssGSEA algorithm and identified the qualified correlation of infiltrated immune cells and *UNC93B1* expression by Pearson correlation coefficient (Bindea et al., 2013; Hänzelmann et al., 2013). A total of 24 subtypes of infiltrating immune cells were involved, including dendritic cells (DCs), activated dendritic cells (aDCs), immature dendritic cells (iDCs), plasmacytoid dendritic cells (pDCs), B cells, T cells, CD8 positive (CD8^+^) T cells, T helper cells, T central memory (Tcm) cells, T effector memory (Tem) cells, T follicular helper (Tfh) cells, T gamma delta (Tgd) cells, Th1 cells, Th17 cells, Th2 cells, regulatory T (Treg) cells, cytotoxic cells, eosinophils, macrophages, mast cells, neutrophils, NK cells, NK CD56 (bright) cells, and NK CD56 (dim) cells. Next, we draw a scatterplot in R by ggplot2 to show a quantitative correlation of *UNC93B1-*expressed level and well-recognized surface markers of specific immune cells (CD14, CD68), which were widely discussed in monocytes/macrophages.

### Statistical analysis

Statistical analysis was carried out based on the R package (https://www.r-project.org/, version 3.6.3). Statistically significant was defined as a two-tailed *p*-value of <0.05. Differences in clinical features based on *UNC93B1* expression were defined by the Kruskal–Wallis tests and the Wilcoxon rank sum tests. The Pearson χ2 test was used to detect differences in clinical features between low and high UNC93B1-expressed AML. The survival curve was plotted using the Kaplan-Meier (KM) method, and the logarithmic rank *p*-value < 0.05 was considered statistically significant. Univariate and multivariate Cox regression analysis were selected to determine prognostic factors. Risk ratios (HR) and 95% confidence intervals (CI) for survival-related genes or events were calculated.

## Results

### High expression of *UNC93B1* and its implicated clinical characteristics in AML

**FIGURE 1:**
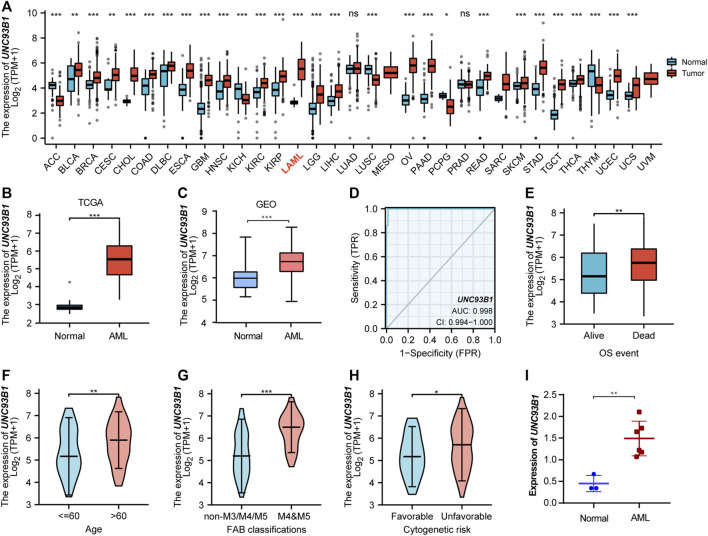
High *UNC93B1* expression was linked to inferior clinical features in AML. **(A)** Level of *UNC93B1* expression in different tumors from TCGA and GTEx database. **(B)** Expression levels of *UNC93B1* in AML (n = 173) and normal samples (*n* = 70). **(C)** Differential expression of *UNC93B1* expression between AML patients from GSE13159 (*n* = 252) and normal control from GSE42519 (*n* = 44). **(D)** Receiver operating characteristic analysis (ROC) of *UNC93B1* in AML. **(E)** Expression levels of *UNC93B1* in different final OS events. **(F)** Association of *UNC93B1* expression and age. **(G)** High expression of *UNC93B1* in M4/M5 leukemia according to French-American-British (FAB) classification. **(H)** High level of *UNC93B1* relative to GAPDH in AML patients (*n* = 6) compared to healthy donors (*n* = 3) using qPCR. **(I)** Association of high *UNC93B1* expression and unfavorable Cytogenetics according to 2017 European Leukemia Net (ELN) genetic risk stratification guidelines of AML. Wilcoxon rank sum test was used between two groups of unpaired samples and Kruskal–Wallis rank sum test was analyzed among multiple groups of samples. ns: *p* ≥ 0.05, *p < 0.05, **p < 0.01, ***p < 0.001.

To explore the expression landscape and potential indication of UNC93B1, the mRNA levels of this molecular in tumors and normal tissues were extracted from TCGA database. Pan-cancer analysis revealed that *UNC93B1* was widely and highly expressed in most malignancies (Figure 1A), especially in AML (*p* < 0.001, Figure 1B). We validated the high expression level of *UNC93B1* in another GEO datasets (GSE13159, *p* < 0.001, Figure 1C).

Intriguingly, UNC93B1 expression distinguished AML from healthy individuals with a predictive power of 0.998 (95% confidence interval, CI = 0.994–1.000), as shown by AUC values analyzed by the ROC curve (Figure 1D). Therefore, we doubted whether *UNC93B1* expression in AML patients is clinically relevant. We surprisingly observed *UNC93B1* expression is linked to final OS events (*p* < 0.01, Figure 1E) and other clinical characteristics (Table 1). The *UNC93B1* expression level was higher in AML patients with older age (age > 60, *p* < 0.01, Figure 1F), non-M3 FAB classification (*p* < 0.05, Supplementary Figure S1A), especially M4/M5 FAB subtype (*p* < 0.001, Figure 1G), in which monocytes were prominent. Unexpectedly, unfavorable cytogenetic risk including intermediate or poor cytogenetic risk, which indicated inferior survival in 2017 European Leukemia Net (ELN) genetic risk stratification had higher level of *UNC93B1* (*p* < 0.05, Figure1H). Moreover, among the favorable cytogenetic risk, upregulated *UNC93B1* was associated with inv (16) (*p* < 0.01, Supplementary Figure S1B), which results in the fusion oncoproteins CBFβ-SMMHC. As we all known, AML subtype M4-with eosinophilia (M4-eo) is classified by AML blasts with inv (16) that have a myelomonocytes with immature basophilic granules (Sangle and Perkins, 2011), which is consistent with our results that *UNC93B1* is higher in M4/M5 FAB subtypes leukemia. Furthermore, we performed quantitative real-time PCR (qRT-PCR) of our clinical samples to confirm that *UNC93B1* were high-expressed in AML-M4/M5 FAB subtypes (*p* < 0.005, Figure 1I). Whereas, there is no association with gender, WBC counts, *FLT3*, *IDH1*, *RAS* and *NPM1* mutation status (Table1). Taken together, high *UNC93B1* expression was closely linked to unfavorable clinical characteristics and deserved further exploration in AML.

**TABLE 1:** Clinical characteristics of AML patients in two groups divided by the level of UNC93B1 expression.

Characteristic	Low expression of *UNC93B1*	High expression of *UNC93B1*	*p*
n	75	76	
Gender, n (%)			0.928
Female	33 (21.9%)	35 (23.2%)	
Male	42 (27.8%)	41 (27.2%)	
Race, n (%)			0.154
Asian	0 (0%)	1 (0.7%)	
Black or African-American	9 (6%)	4 (2.7%)	
White	64 (43%)	71 (47.7%)	
Age, n (%)			**0.025**
≤60	51 (33.8%)	37 (24.5%)	
>60	24 (15.9%)	39 (25.8%)	
WBC count(x10^9/L), n (%)			0.140
≤20	43 (28.7%)	34 (22.7%)	
>20	31 (20.7%)	42 (28%)	
BM blasts (%), n (%)			0.920
≤20	29 (19.2%)	31 (20.5%)	
>20	46 (30.5%)	45 (29.8%)	
Cytogenetic risk, n (%)			0.354
Favorable	18 (12.1%)	13 (8.7%)	
Intermediate	36 (24.2%)	46 (30.9%)	
Poor	19 (12.8%)	17 (11.4%)	
FAB classifications, n (%)			**< 0.001**
M0	10 (6.7%)	5 (3.3%)	
M1	18 (12%)	17 (11.3%)	
M2	26 (17.3%)	12 (8%)	
M3	11 (7.3%)	4 (2.7%)	
M4	5 (3.3%)	24 (16%)	
M5	2 (1.3%)	13 (8.7%)	
M6	1 (0.7%)	1 (0.7%)	
M7	1 (0.7%)	0 (0%)	
Cytogenetics, n (%)			**0.032**
Normal	30 (22.2%)	39 (28.9%)	
+8	5 (3.7%)	3 (2.2%)	
del (5)	1 (0.7%)	0 (0%)	
del (7)	4 (3%)	2 (1.5%)	
inv (16)	1 (0.7%)	7 (5.2%)	
t (15; 17)	7 (5.2%)	4 (3%)	
t (8; 21)	7 (5.2%)	0 (0%)	
t (9; 11)	0 (0%)	1 (0.7%)	
Complex	13 (9.6%)	11 (8.1%)	
FLT3 mutation, n (%)			0.846
Negative	51 (34.7%)	51 (34.7%)	
Positive	21 (14.3%)	24 (16.3%)	
IDH1 R132 mutation, n (%)			0.077
Negative	64 (43%)	72 (48.3%)	
Positive	10 (6.7%)	3 (2%)	
IDH1 R140 mutation, n (%)			1.000
Negative	67 (45%)	70 (47%)	
Positive	6 (4%)	6 (4%)	
IDH1 R172 mutation, n (%)			0.238
Negative	71 (47.7%)	76 (51%)	
Positive	2 (1.3%)	0 (0%)	
RAS mutation, n (%)			0.719
Negative	71 (47.3%)	71 (47.3%)	
Positive	3 (2%)	5 (3.3%)	
NPM1 mutation, n (%)			0.758
Negative	59 (39.3%)	58 (38.7%)	
Positive	15 (10%)	18 (12%)	

The two-tailed *p*-value of <0.05 was considered to be statistically significant and highlighted in bold format.

### High *UNC93B1* expression predicts worse prognosis in AML

**FIGURE 2:**
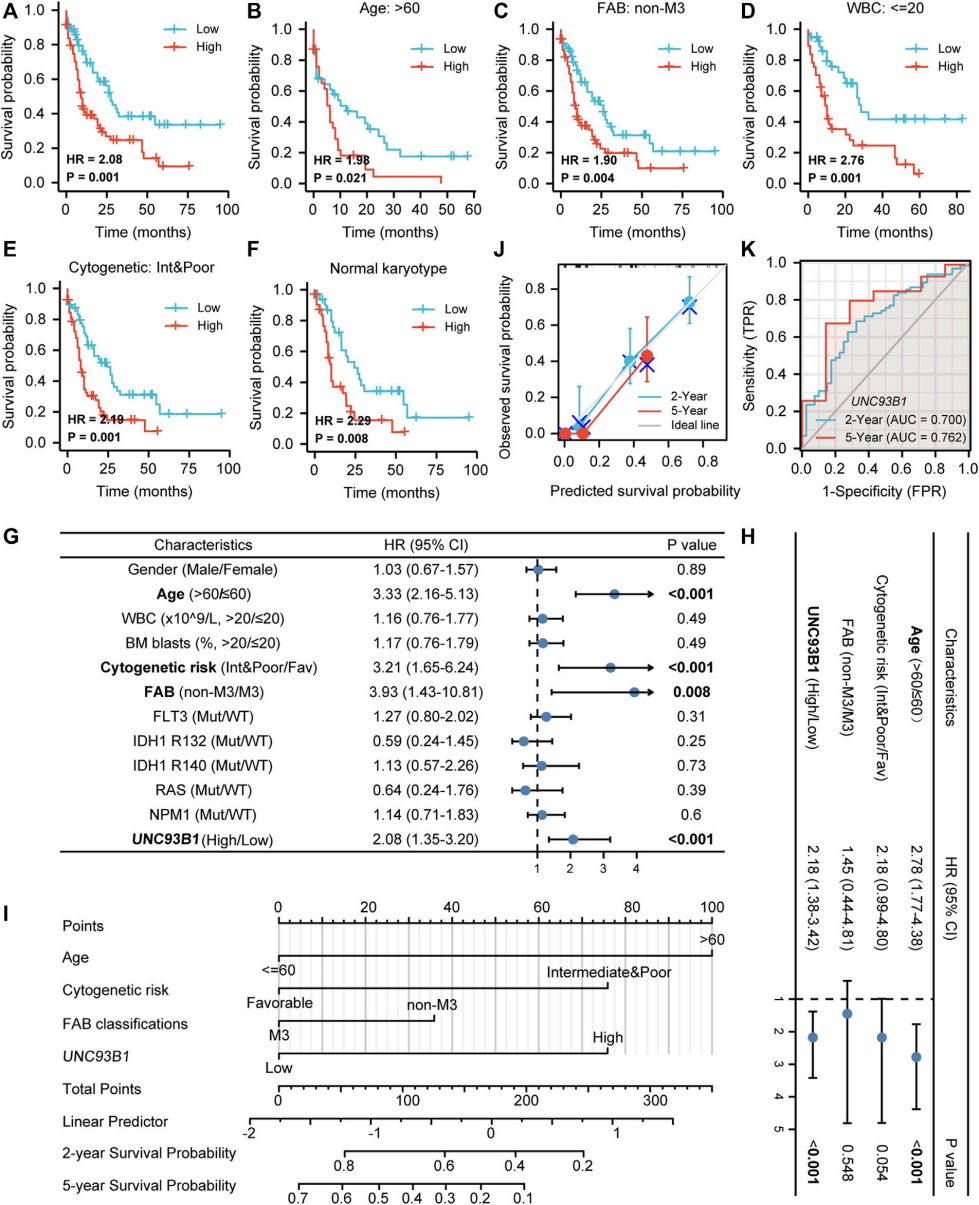
Unfavorable prognostic value of high *UNC93B1* expression in AML. **(A)** KM curve analysis of overall survival (OS) between high and low *UNC93B1* expression Groups divided by median expression value of *UNC93B1* in AML. **(B–F)** Prognostic value of *UNC93B1* expression in each subgroup divided by clinical features. **(B)** AML patients with age > 60 years old, **(C)** AML patients with all other subtypes except for M3 according to French-American-British (FAB) classification, **(D)** AML patients with WBC counts in peripheral blood <20 x10^9/L, **(E)** AML patients with unfavorable (intermediate and poor) Cytogenetic risk classification according to 2017 European Leukemia Net (ELN) genetic risk stratification guidelines of AML, **(F)** AML patients with Normal karyotype. **(G)** Forest plot of Univariate analyses of OS **(H)** Forest plot of Multivariate analyses of OS. **(I)** Nomogram model integrates *UNC93B1* and other prognostic factors in AML. **(J)** Calibration curve of nomogram. **(K)**The Receiver operating characteristic analysis (ROC) by *UNC93B1* in predicting 2-year and 5-year OS of AML. Mut: mutation; wt: wild type; Int: intermediate; Fav: favorable; non-M3 including M0, M1, M2, M4, M5, M6 and M7 of FAB classification.

To explore the prognostic value of UNC93B1 in AML, we draw the overall survival (OS) curve using the Kaplan-Meier method. Patients with higher levels of *UNC93B1* than the median expression value had a worse overall survival (HR = 2.08, *p* = 0.001, Figure 2A). Furthermore, we rationally proposed *UNC93B1* level can also distinctly reflect survival outcomes of different subgroups, and the analysis showed higher *UNC93B1* level had a more pronounced shortens of survival in AML patients with older age (age > 60, HR: 10.98, *p* = 0.021, Figure 2B), non-M3 FAB subtype (HR: 1.90, *p* = 0.004, Figure 2C), WBC counts in peripheral blood ≤ 20 x10^9/L (HR: 2.76, *p* = 0.001, Figure 2D), and unfavorable cytogenetic risk (including Intermediate cytogenetic risk and Poor cytogenetic risk, HR = 2.49, *p* = 0.001, Figure 2E). Additionally, higher expression of *UNC93B1* also a poor survival factor in AML patients with normal karyotype (HR: 2.29, *p* = 0.008, Figure 2F), which is predominant in intermediate-risk group (Lin et al., 2017). Interestingly, we found high expression of UNC93B1 just linked to worse prognosis in AML patients without FLT3 (*p* < 0.05, Supplementary Figure S2A), IDH1 (*p* < 0.05, Supplementary Figures S2B–D) or NRAS (*p* < 0.05, Supplementary Figure S2H) mutation, once acquired mutations of these genes, *UNC93B1* expression level had no effects on patient’s survival outcome (*p* > 0.05, Supplementary Figures S2E–G). Meanwhile, there is a significantly negative correlation between *UNC93B1* level and OS of both subgroups divided by BM blasts and NPM1 (*p* < 0.05, Supplementary Figures S2I–L).

To further identify the indicated prognosis of UNC93B1 in AML and other survival-related factors, we performed univariate and multivariate Cox regression analyses (Supplementary Table S3). Univariate analysis indicated that older age (age > 60, HR: 3.33, 95% CI: 2.16–5.13, *p* < 0.001), unfavorable cytogenetic risk (including Intermediate cytogenetic risk and Poor cytogenetic risk, HR: 3.21, 95% CI: 1.65–6.24, *p* < 0.001), non-M3 FAB subtype (HR: 3.93, 95% CI: 1.43–10.81, *p* = 0.008) and higher *UNC93B1* levels (HR: 2.08, 95% CI: 1.35–3.20, *p* < 0.001) predicted poor survival (Figure 2G). Multivariate Cox regression analyses showed that except for age > 60 (HR: 2.78, 95% CI: 1.77–4.38, *p* < 0.001), expression level of *UNC93B1* (HR: 2.18, 95% CI: 1.38–3.42, *p* < 0.001) was the only factor which have clinically meaningful effects to overall survival (Figure 2H). Thus, *UNC93B1* is an independent prognostic factor found in both univariate and multivariate analysis.

Additionally, a nomogram model was built using age, FAB subtype, cytogenetic risk and *UNC93B1* gene expression, which were independent prognostic factors in univariable analysis, to provide the model’s quantitative predictive power for survival outcomes in AML patients (Figure 2I). The predictive OS concordance index (C-index) was 0.729 (95% CI: 0.701–0.756, *p* < 0.001, Figure 2I), indicating that these four univariable factors had a certain predictive accuracy for OS. Calibration analysis was performed to further assess the predictive value of the nomogram model, in which the calibration curves showed the agreement between the predicted OS and the actual proportion of OS at different time points, and the 5-year median survival rate is sharply decreased compared to 2-year median survival rate (Figure 2J), probably in part due to the higher relapse rate of AML within 2 years clinically. Thus, this nomogram model is well calibrated and had an accuracy to predict the patients’ clinical outcomes. Finally, time-dependent analysis of sensitivity, specificity, and ROC curve was constructed to evaluate *UNC93B1* for predicting survival outcome at 2 years or 5 years, and the cumulative 2-year survival rate is 43.7% (2-year AUC = 0.70, 95% CI: 0.60–0.80), while the cumulative 2-year survival rate was dramatically decreased to 21.9% (5-year AUC = 0.76, 95% CI:0.59–0.94, Figure 2K), which is consistent with clinical data. In summary, the survival-predictive model constructed here is significant for AML and *UNC93B1* served as an independent prognostic factor for poorer outcome.

### Differentially expressed gene analysis and functional signaling pathway enrichment in AML

**FIGURE 3:**
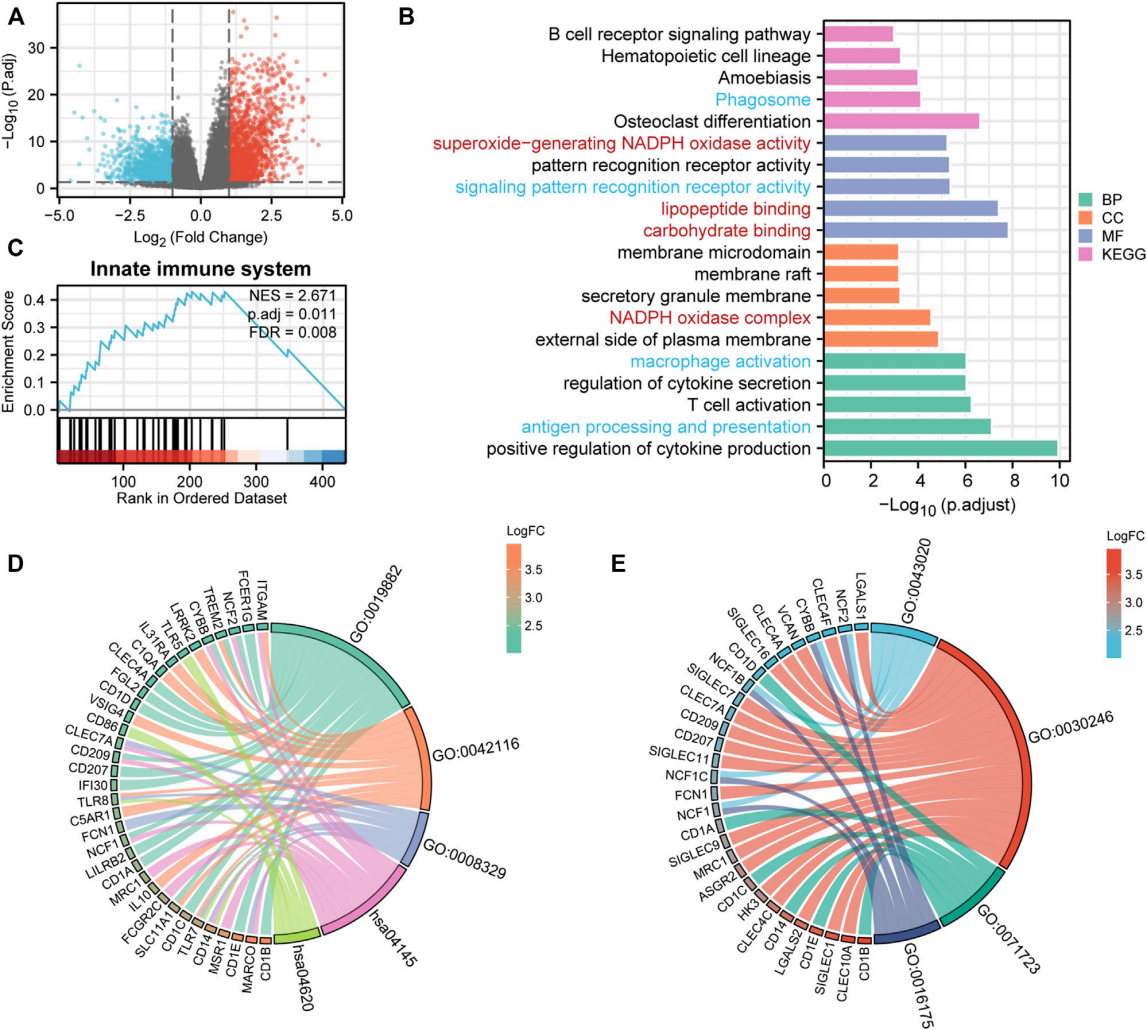
DEG analysis and Functional enrichment analyses of DEGs. **(A)** Volcano plot of DEGs. **(B)** GO and KEGG pathway of up-regulated DEGs. Red context represents innate-immune signaling sets and blue means metabolic-related pathway sets. **(C)** Enriched in innate immune system by GSEA analysis. immune system. **(D–E)** Interactive analysis of enriched pathways in GO/KEGG and corresponding genes. **(D)** Innate-immune signaling sets and the corresponding genes. GO:0019882, antigen processing and presentation; GO:0042116, macrophage activation; GO:0008329, signaling pattern recognition receptor activity; hsa04145, Phagosome; hsa04620, Toll-like receptor signaling pathway. **(E)** Metabolic-related signaling sets and the corresponding genes. GO:0043020, NADPH oxidase complex; GO:0030246, carbohydrate binding; GO:0071723, lipopeptide binding; GO:0016175, superoxide-generating NADPH oxidase activity.

To explore the potential mechanisms of *UNC93B1* in leukemogenesis, we first identified the DEGs between two AML populations divided by high and low *UNC93B1* expression. In total, 614 DEGs (|log2(FC)| >2 and adjusted *p* value < 0.05) were obtained and shown in volcano plots (Figure 3A; Supplementary Table S4), including 342 upregulated genes and 272 downregulated genes. To elucidate the intracellular signaling pathways influenced by the DEGs, we performed GO and KEGG analysis. Firstly, we investigated the biological function of the up-regulated DEGs, the top five of BP enrichment items, CC enrichment items, MF enrichment items as well as top 5 KEGG pathways were obtained from GO annotation (Figure 3B; Supplementary Table S5). We can find two main signaling sets were enriched, one set is immune-related signaling, especially the innate immune associated pathways, such as antigen processing and presentation, macrophage activation, pattern recognition receptor activity, Phagosome and Toll-like receptor signaling pathway were significantly enriched (Figure 3B; Figure 3D; Supplementary Table S5). Another notable signaling cascade was metabolism-related set, including NADPH oxidase complex, carbohydrate binding, lipopeptide binding, superoxide-generating NADPH oxidase activity (Figure 3B; Figure 3E; Supplementary Table S5). Moreover, to better understand the mutual connection among genes and the aforementioned two main signaling sets, we performed an interactive analysis of two main enriched signaling sets and its corresponding genes (Figure 3D-E). We discovered that *CD14*, *TLR8*, *TLR7*, *ITGAM*, *NCF2*, *TREM2*, *CYBB*, *CLEC7A*, *CD209*, *NCF1*, *SLC11A1* and *MARCO* shared by at least two pathways in innate immune-related sets (Figure 3D); while *NCF2*, *CYBB*, *NCF1B*, *NCF1C* and *NCF1* were commonly enriched in metabolism-related sets (Figure 3E).

To validate the activation of innate immune signaling in *UNC93B1*-high expressed AML, we next use GSEA analysis to investigate the enrichment pathway, and finally, we found four significantly activated pathways, which were innate immune system (NES = 2.67, *p* = 0.01, Figure 3C), immunoregulatory interactions between a lymphoid and a non-lymphoid cell (NES = 2.60, *p* = 0.01, Supplementary Figures S3A), neutrophil degranulation (NES = 2.23, *p* = 0.01, Supplementary Figures S3B) and adaptive immune system (NES = 2.94, *p* = 0.01, Supplementary Figure S3C; Supplementary Table S6). So, we assumed that immune system, especially innate immune signaling might play an important role in AML with high level of *UNC93B1.*

### Identification of hub genes connected to *UNC93B1*

**FIGURE 4:**
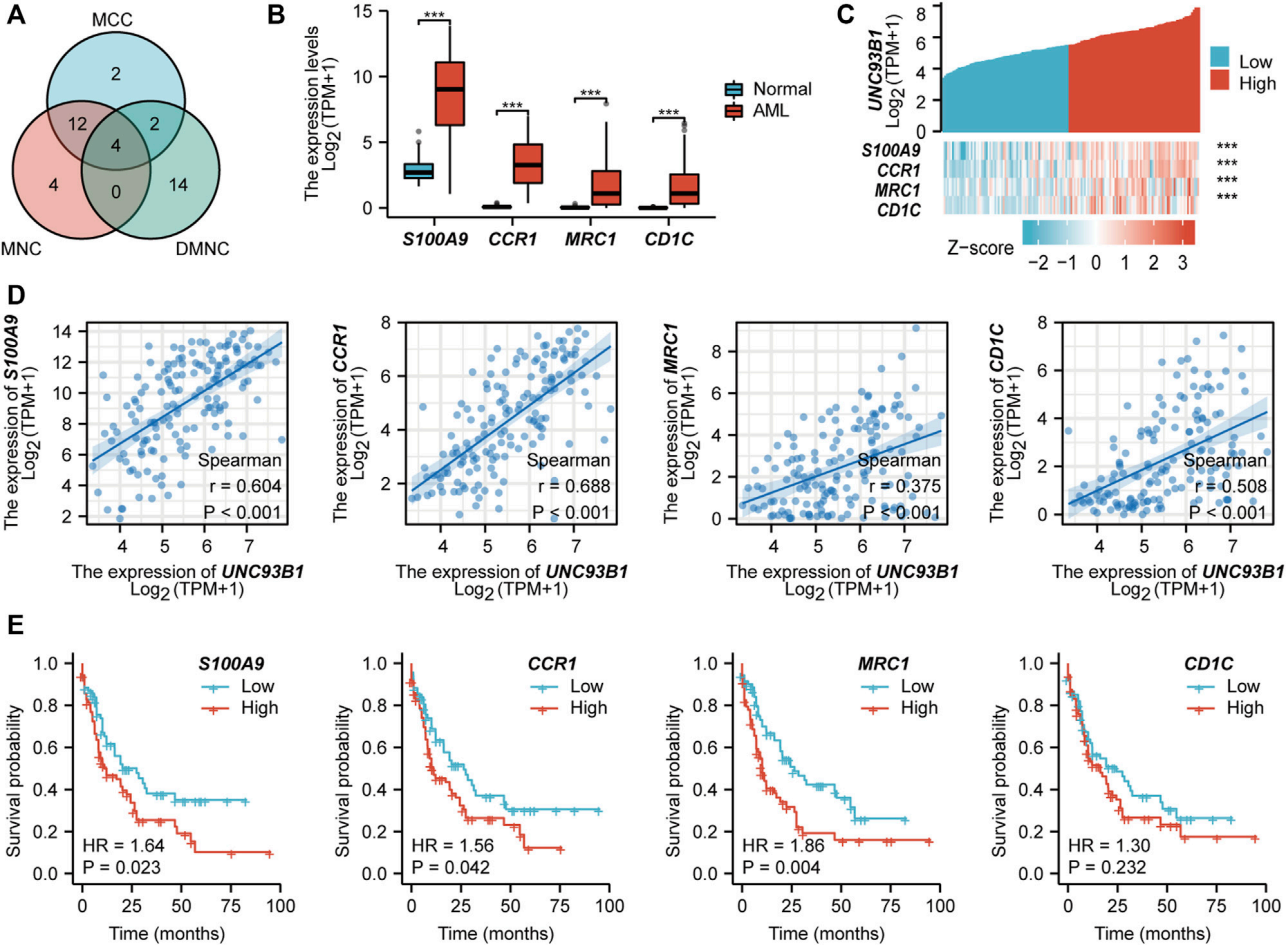
PPI network construction and clinical significance of hub genes. **(A)** Venn diagram of hub genes obtained from three different algorithms. **(B)** Expression of common hub genes (*S100A9*, *CCR1*, *MRC1*, *CD1C*) in AML(*n* = 173) and normal individuals(*n* = 70). **(C)** Co-expression pattern of *UNC93B1* and four hub genes. **(D)** Quantified expression correlation of *UNC93B1* and four hub genes. **(E)** Prognostic value of hub genes in AML. (*p < 0.05, **p < 0.01, ***p < 0.001).

To determine possible proteins directly interact with UNC93B1, protein-protein interaction (PPI) analyses were used. We calculated all of the 342 up-regulated genes and the top 20 hub genes were obtained by the maximal clique centrality (MCC, Supplementary Figures S4A), maximum neighborhood component (MNC, Supplementary Figures S4B), and density of maximum neighborhood component (DMNC, Supplementary Figures S4C) algorithms respectively. Clearly, four common hub genes (*S100A9*, *CCR1*, *MRC1* and *CD1C*) shared from the aforementioned three gene lists (Figure 4A), and all of these four genes are related to innate immune response. To gain further deep insight in these molecules, we next turn to interrogate the expression and survival outcome of these four genes. All hub genes were upregulated in AML patients compared to healthy individuals (*p* < 0.05, Figure 4B). Furthermore, increased expression of *S100A9*, *CCR1* and *MRC1* were also associated with poor survival in AML (*p* < 0.05, Figure 4E). Additionally, *UNC93B1* expression positively correlates with *S100A9*, *CCR1*, *MRC1* and *CD1C* (*p* < 0.001, Figure 4C), and the detailed co-expression pattern were shown in Figure 4D.

### Correlation analysis between *UNC93B1* and immune cell infiltration

**FIGURE 5:**
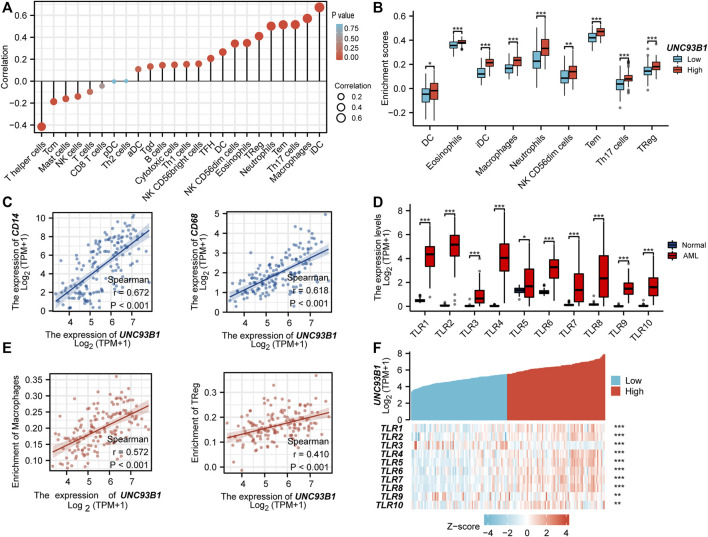
UNC93B1 gene expression related to innate immune cell infiltration and innate-immune related molecules. **(A)** Correlation of UNC93B1 expression and 24 kinds of infiltrated immune cells. **(B)** High UNC93B1 expression enriched more immune cells, especially innate immune cells. **(C)** Positive correlation of UNC93B1 expression level with CD14 and CD68. **(D)** High expression of TLR family in AML (*n* = 173) compare with normal individuals (*n* = 70). **(E)** Positive correlation of UNC93B1 expression level with macrophages and regulatory T cells (Tregs). **(F)** Expression correlation between UNC93B1 and Toll-like receptors (TLRs) genes in AML. (r means Spearman’s correlation coefficient. *p < 0.05, **p < 0.01, ***p < 0.001.)

To investigate the association between *UNC93B1* expression and AML-infiltrating immune cells, we studied a total of 24 immune cell subtypes. The results displayed that AML with high expression of *UNC93B1* had a significant trend to be infiltrated by dendritic cells (DC), immature dendritic cells (iDC), Macrophages, Neutrophils, Eosinophils, NK CD56(dim) cells, Effective Memory T Cell (Tem), Th17 cells and Treg cells (Figure 5A), most of which were responsible for innate immune system instead of adaptive immune. The refined details of a quantified analysis of *UNC93B1* and infiltrated innate immune cells (DC, iDC, macrophages, neutrophils, Eosinophils and NK CD56dim cells) by Spearman’s correlation are shown in Figure 5B. More strikingly, we found the level of *UNC93B1* expression was positively corelated with macrophages (r = 0.572, *p* < 0.001), and regulatory T cells (Tregs, *r* = 0.41, *p* < 0.001) in AML (Figure 5E), which were responsible for suppressing functional T cell activation and regulating immune systems. We also discovered *UNC93B1* expression levels positively correlated with CD14 (r = 0.672, *p* < 0.001, Figure 5C), a surface antigen that is preferentially expressed on monocytes and a molecule associated to shorter overall survival and lower complete remission rate in AML (Solary et al., 1992; Bradstock et al., 1994). CD68, generally represented macrophages and associated with a lower complete remission in AML (Liu et al., 2022), were also found to be higher in *UNC93B1*-high expressed AML patients (*r* = 0.618, *p* < 0.001, Figure 5C), which further validated the role of innate immune cells in AML pathogenesis and therapy response. TLRs, the most important family of receptors in the early to middle stages of innate immunity response, play a crucial role in recognizing microorganisms and augmenting the inflammatory mediators (Shey et al., 2010; Takeuchi and Akira, 2010; Trejo-de la et al., 2014; Schnetzke et al., 2015), were all extremely upregulated in AML (*p* < 0.05, Figure 5D), consistent with dysfunctional innate immune response in AML. Unsurprisingly, the level of *UNC93B1* was positively and significantly correlated with *CD14*, *CD68*, and almost all of TLRs except for *TLR3* (*p* < 0.001, Figure 5C; Figure 5F).

### UNC93B1 expression in AML is associated with innate immune and metabolic process

**FIGURE 6:**
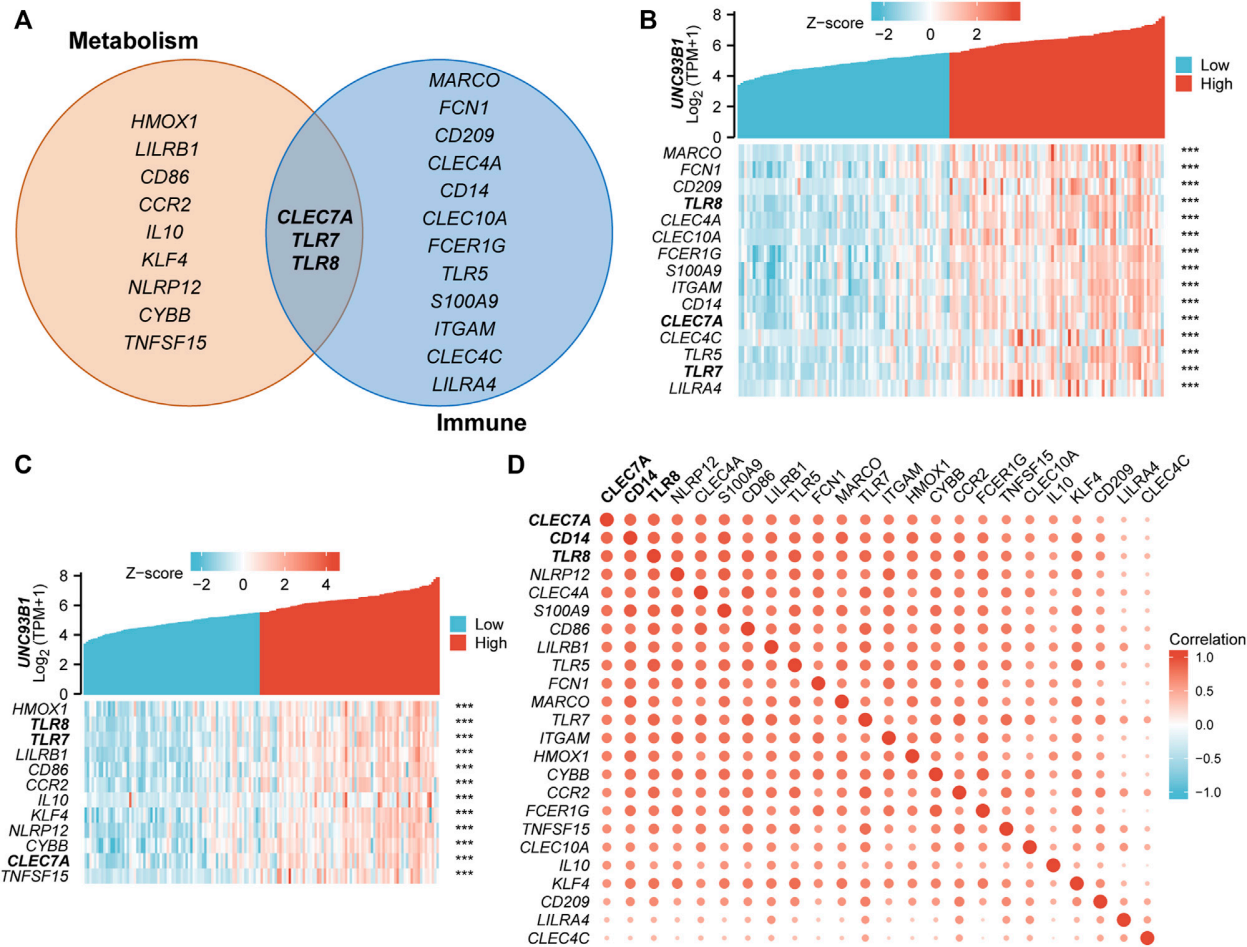
Association of UNC93B1 with innate immune and metabolic process. **(A)** Venn diagram of genes enriched in innate immune response-activating signal transduction pathway (right) and cytokine metabolic process signaling (left). **(B)** Co-expression heatmap of UNC93B1 and innate immune response-activating signal transduction related genes. **(C)** Co-expression heatmap of UNC93B1 and cytokine metabolic process related genes. **(D)** Positive correlation of genes’ expression corresponding to innate immune response-activating signal transduction and cytokine metabolic process.

To refine the potential mechanism of *UNC93B1* on leukemogenesis, we quantified the correlation of expression between *UNC93B1* and the genes related to innate immune response-activating signal transduction (Figure 6B) and cytokine metabolic process (Figure 6C), both were significantly enriched in GO/KEGG (Figure 3B). Gene lists related to each pathway were shown in Figure 6A, and *CLEC7A*, *TLR7*, *TLR8* played important roles in both signaling. C-Type Lectin Domain Family 7 Member A (*CLEC7A*), encoding a glycoprotein that is a small type II membrane receptor with extracellular C-type lectin-like domain folding and a cytoplasmic domain with an activated motif based on immune receptor tyrosine. Gene Ontology (GO) annotations associated with this gene include carbohydrate binding and pattern recognition receptor activity, which is consistent with the mutually shared genes in aforementioned immune and metabolic pathway. What’s more, TLR7 and TLR8 predominantly expressed in dendritic cells, TLR8 is also expressed in monocytes, macrophages, neutrophils, and Tregs (Gorden et al., 2005; Peng et al., 2005). TLR7 activation triggers the production of interferon-α (Gorden et al., 2005), and TLR8 activation leads to the production of immunomodulatory cytokines, such as interleukin-12, interleukin-18, and pro-inflammatory cytokines, such as tumor necrosis factor alpha and interleukin-1β (Cristescu et al., 2018), which is associated with leukemic microenvironment and immune tolerance (Pyzer et al., 2017). Therefore, the high expression of TLRs on leukemia cells and the consequences of TLR activation might be a potentially therapeutic target.

There have been multiple studies that indicate innate immune cells undergo substantial metabolic reprogramming following activation (Angiari et al., 2020). Meanwhile, various metabolic enzymes involved in glycolysis and mitochondrial metabolic pathways have been found to play a vital role in influencing innate immune cell function (Li et al., 2019). So, we supposed the coupling of metabolic process and innate immune responses play an essential role in the AML development, especially in the patients with high-expressed *UNC93B1*. And we draw the correlation heat maps of the key regulators in metabolic process and innate immune pathways (Figure 6D), which demonstrated obvious co-expression patterns. Therefore, we conclude that immunometabolism has taken on a different meaning in AML by unknown immune-metabolism interactions, for example, contributions of key metabolic factors contribute to immune cell development or fate; in turn, responses of immune cells resulted in metabolic reprogramming. Altogether, metabolic system and innate immune affected AML patients with aberrantly high-expressed *UNC93B1* through combined strategies.

### UNC93B1 is preferentially high expressed on monocytic AML and associated with losing expression of venetoclax target BCL2

**FIGURE 7:**
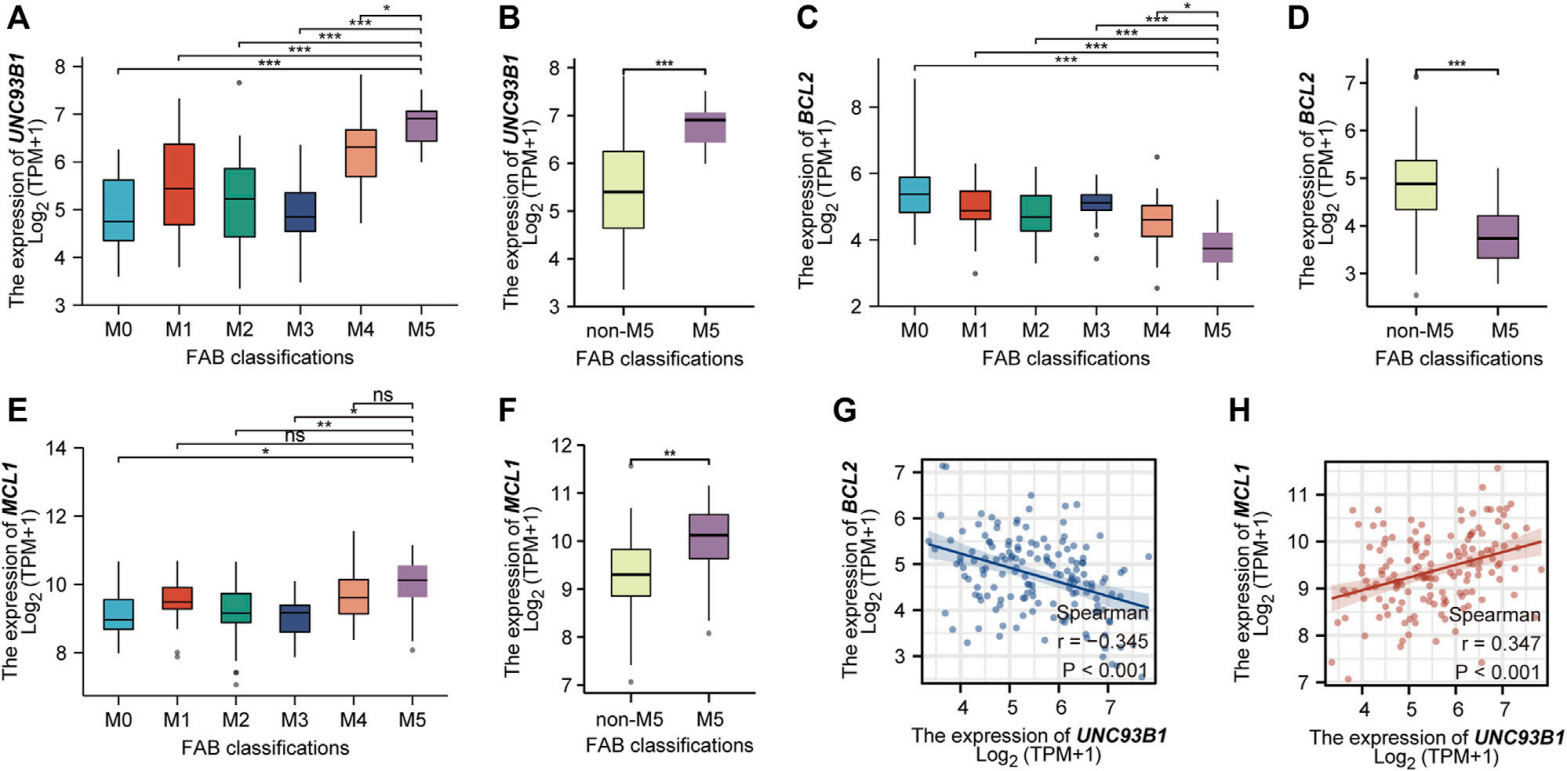
High expression of *UNC93B1* in FAB-M5 AML and association with losing expression of venetoclax target BCL2. **(A)** The expression level of *UNC93B1* in different FAB subtypes (M0-M5) of AML. M6 and M7 were excluded because of small numbers (*n* ≤ 3). **(B)** The expression level of *UNC93B1* in FAB-M5 AML and non-M5 AML. non-M5 including M0, M1, M2, M3, M4, M6 and M7 of FAB classification. **(C)** The expression level of *BCL2* in different FAB subtypes (M0-M5) of AML. **(D)** The expression level of *BCL2* in FAB-M5 AML and non-M5 AML. **(E)**The expression level of *MCL1* in different FAB subtypes (M0-M5) of AML. **(F)** The expression level of *MCL1* in FAB-M5 AML and non-M5 AML. **(G)** The negative correlation of *UNC93B1* expression and *BCL-2* expression in AML. **(H)** The positive correlation of *UNC93B1* expression and *MCL1* expression in AML. (r means Spearman’s correlation coefficient. Wilcoxon rank sum test was used between two groups of unpaired samples, ns: *p* ≥ 0.05, *p < 0.05, **p < 0.01, ***p < 0.001.)

As we show in this work, *UNC93B1* is high-expressed in AML, especially in M4/M5 (Figure 1G). Recent studies have shown that AML hierarchy composition as a determinant of response to targeted therapy (Zeng et al., 2022), and Monocytic AML (M5) is intrinsically resistant to venetoclax + azacytidine (Pei et al., 2020). We then further analyzed the expression of *UNC93B1* in AML and found that the level of UNC93B1 is significantly higher in FAB-M5 patients than any other FAB-subtype patient (*p* < 0.05, Figure 7A-B), which is consistent with the positive correlation expression of *UNC93B1* and *CD14*, *CD68* shown in Figure 5C. As we demonstrated in Figure 3B, another signaling enriched by UNC93B1 were metabolic pathways, and AML blasts might switch from BCL2-dependence to MCL1-dependence to drive energy metabolism under specific circumstances (Pei et al., 2020), we next focused on expression of BCL2 and MCL-1 in AML. Contrary to the increasing expression of *UNC93B1* in FAB-M5 AML, *BCL2* expression decreased remarkably in FAB-M5 AML (*p* < 0.05, Figures 7C–D). However, *MCL1* presented similar expression pattern to *UNC93B1* in AML, tended to be high-expressed in FAB-M5 (*p* < 0.05, Figures 7E–F). To clear the quantitative expression correlation of *UNC93B1*, *BCL2* and *MCL1*, we performed Spearman Rank Correlation Analysis, and as expected, we revealed significantly negative correlation of *UNC93B1* expression and *BCL2* expression (*r* = −0.345, *p* < 0.001, Figure 7G), meanwhile, consistent with the previous results, *MCL-1* expression is positively related to *UNC93B1* level in AML (*r* = 0.347, *p* < 0.001, Figure 7H). Given that venetoclax (VEN) is an inhibitor specific to BCL2 and some studies have discovered that the level of BCL2 expression is closely related to VEN response *in vitro* (Souers et al., 2013; Pan et al., 2014), we hypothesized high-UNC93B1 AML might be resistant to venetoclax, and selective MCL1 inhibitor, such as VU661013 (Ramsey et al., 2018), alone or in combination with AZA might be effective in venetoclax-resistant AML and high-*UNC93B1* AML. Together, these data indicated that high-*UNC93B1* expressed AML cells shows greater dependence on MCL1 than BCL2 for survival, and *UNC93B1* might be a biomarker to predicting drug response in acute myeloid leukemia.

## Discussion

Acute myeloid leukemia (AML) is a heterogeneous group of hematologic malignancies with low survival rates and with most patients eventually relapsing and dying of progressive disease. Resistance to chemotherapy and relapse after HSCT were major obstacles in the cure of AML patients, and is often attributed to the immune escape of AML blasts (Dermime et al., 1997; Masuda et al., 2007; Vago et al., 2009; Stölzel et al., 2012; Dama et al., 2019). Compared to the rapid development of immunotherapies in solid tumors, PD1/PD-L1 antibodies showed limited clinical activity in AML (Zhang et al., 2009; Zhou et al., 2010; Zhou et al., 2011), we still have a long way to develop immunotherapies for myeloid leukemia. Recent lines of investigation suggest that innate immunity might play an important role in hematopoietic malignancies (Tettamanti et al., 2022).

The ER membrane protein uncoordinated 93 homolog B1 (UNC93B1) plays an important role in regulating intracellular TLR signaling, which is vital in innate immune system. Few studies reported that UNC93B1 had a relation with some specific type of solid tumor (Wagai et al., 2019; Zhao et al., 2019). However, it is remains elusive whether UNC93B1 has an impact on AML.

We first investigated the association between *UNC93B1* expression and clinical features of AML. Surprisingly, *UNC93B1* expression observed in AML was aberrantly up-regulated, which correlated with adverse clinical characteristics and poor survival. Next, we used the integrated bioinformatics analysis of DEGs to explore the potential pathogenic mechanisms of UNC93B1. GO/KEGG and GSEA analyses consistently pointed to dysregulated innate immune signaling in AML patients with high-*UNC93B1* expression. Therefore, we proposed that *UNC93B1* is involved in innate immune system, especially TLR signaling pathway, which may contribute to the poor outcomes in AML.

In addition, we demonstrated that S100A9, CCR1, MRC1 and CD1C act as hub genes for high-UNC93B1 AML, and three (S100A9, CCR1, MRC1) of them were also related to poor survival of AML. S100A9 is a calcium- and zinc-binding protein that plays an essential role in regulating inflammatory processes and immune response. CCR1, Macrophage Inflammatory Protein 1-Alpha Receptor, participated in recruiting the effector immune cells to the inflammatory site. MRC1, Macrophage Mannose Receptor 1-Like Protein 1, involve in mediating the endocytosis of glycoproteins by macrophages. CD1C, T-Cell Surface Glycoprotein CD1c, involved in Dendritic Cells Developmental Lineage Pathway and Innate Immune System. Thus, all hubs we obtained here were innate immune related genes. Furthermore, high levels of innate immune cells infiltration and wide expression of TLR signaling genes also validated innate immune activation, which further support our hypothesis.

Additionally, we found the higher expression of *UNC93B1*, the more innate immune cells infiltrated such as macrophages and dendric cells. Instead of activating functional effector T cells, *UNC93B* tended to recruit regulatory T cells, which were responsible for the initiation and development of acute and chronic leukemia (Moon et al., 2011; Bachireddy et al., 2015; Wan et al., 2020), and mediated immune escape of myeloblast in *de novo* AML (Wan et al., 2020). It is widely believed that metabolism and immune cell function are related (Brown and Byersdorfer, 2020), and innate immune training is associated with metabolic reprogramming (Bandyopadhaya et al., 2016). So, we next investigated the cross-talk between innate immune and metabolic process, unexpectedly, we found they were positively correlated. Finally, we supposed that a large fraction of immune cells infiltrated in AML are from the innate arm of the immune system. These classical innate immune cells (macrophages, DCs, neutrophils, Basophils, Eosinophils, Mast cells and NK cells) (Bandyopadhaya et al., 2016) offers an alternative immunotherapeutic option to attack tumor cells, and targeting TLRs or metabolism signaling dysregulated by UNC93B1 might be an effective treatment too. We believed that more tailored immunometabolism-therapeutic strategies for the future of AML treatment deserved to be deeply explored.

A selective inhibitor of BCL-2, Venetoclax (VEN), has shown the advancement in the treatment of AML patients, especially in older patients (Konopleva et al., 2016). The combination of hypomethylating drugs with venetoclax had been approved by FDA for *de novo* AML patients over the age of 75 or who are not suitable for intensive chemotherapy (Konopleva et al., 2016). However, the widespread use of Venetoclax presented us with new challenges to drug resistance, especially in patients with relapsed/refractory AML (DiNardo et al., 2018; Maiti et al., 2021). Here, we show that UNC93B1 is associated with Venetoclax resistance in AML due to BCL2 loss and discuss the potential of MCL-1 inhibitors to overcome the resistance.

Our results provide the first proof-of-principle that *UNC93B1* is an innate immune related gene, which can serve as prognostic and therapeutic biomarkers. However, several limitations exist with our studies. Firstly, we investigated the diagnostic and prognostic effect of UNC93B1 from the public TCGA AML datasets, which needs to be validated in larger clinical cohorts in the future. Secondly, the interaction and underlying molecular mechanisms between UNC93B1 and the innate immune response lack biological validation. Last but not least, the significance of UNC93B1 in predicting venetoclax resistance and the simultaneous loss of *BCL2* in high-UNC93B1 monocytic AML subpopulations need further experiments. Therefore, clinical trials of TLR inhibitors, MCL-1 inhibitors, innate immune cell therapy and further functional validation in the laboratory are required to overcome the above shortcomings.

## Conclusion

We identified a novel gene (*UNC93B1*) for AML, which serve as a critical candidate of leukemic biomarker and prognostic predictor. Importantly, innate immune response and metabolism signaling dysregulated by UNC93B1 deserved further explorations to define its therapeutic potential, and immuno-metabolism combined strategies might hold great prospect for various applications in AML.

## Data Availability

The original contributions presented in the study are included in the article/Supplementary Material, further inquiries can be directed to the corresponding author.

## Supplementary material

SUPPLEMENTARY FIGURE S1 Association of *UNC93B1* expression and clinical characteristics.**(A)** High level of *UNC93B1* in non-M3 leukemia compared to M3 leukemia according to French-American-British (FAB) classification. **(B)** Differential expression of *UNC93B1* according to cytogenetics. ns: *p* ≥ 0.05, **p* < 0.05, ***p* < 0.01, ****p* < 0.001.

SUPPLEMENTARY FIGURE S2 Survival analysis of *UNC93B1* in different subgroups based on clinical characteristics.**(A)** AML patients without FLT3 mutation. **(B–D)** AML patients without IDH1 mutation. **(E)** AML patients with FLT3 mutation. **(F)** AML patients with IDH1 R132 mutation. **(G)** AML patients with IDH1 R140 mutation. **(H)** AML patients without RAS mutation. **(I)** AML patients with BM blasts (%) ≤ 20%. **(J)** AML patients with BM blasts (%) > 20%. **(K)** AML patients without NPM1 mutation. **(L)** AML patients with NPM1 mutation.

SUPPLEMENTARY FIGURE S3 GSEA analysis of DEGs.**(A)** Enriched in immunoregulatory interactions between a lymphoid and a non-lymphoid cell, **(B)** Enriched in neutrophil degranulation, **(C)** Enriched in adaptive immune system.

SUPPLEMENTARY FIGURE S4 PPI network construction.**(A-C)** Top 20 hub genes selected from the PPI network of up-regulated encoding DEGs calculated by **(A)** MCC, **(B)** MNC, **(C)** DMNC algorithms respectively. MCC, maximal clique centrality; MNC, maximum neighborhood component; DMNC, density of maximum neighborhood component.

SUPPLEMENTARY TABLE S1 Clinical information of AML patients involved in this study.

SUPPLEMENTARY TABLE S2 Primer sequence in qPCR.

SUPPLEMENTARY TABLE S3 Univariate and multivariate Cox regression analyses of survival-related factors in AML.

SUPPLEMENTARY TABLE S4 Differentially expressed genes (DEGs) identified between *UNC93B1*-high AML and *UC993B1*-low AML.

SUPPLEMENTARY TABLE S5 GO and KEGG pathway analysis of up-regulated genes in *UNC93B1*-high AML.

SUPPLEMENTARY TABLE S6 Gene Set Enrichment Analysis.
