# Supplementary figures and images for: High-expression of the innate-immune related gene UNC93B1 predicts inferior outcomes in acute myeloid leukemia
Source: Front Genet. 2023 Jan 18;14:1063227. doi: 10.3389/fgene.2023.1063227 (PMC9891309; doi:10.3389/fgene.2023.1063227)

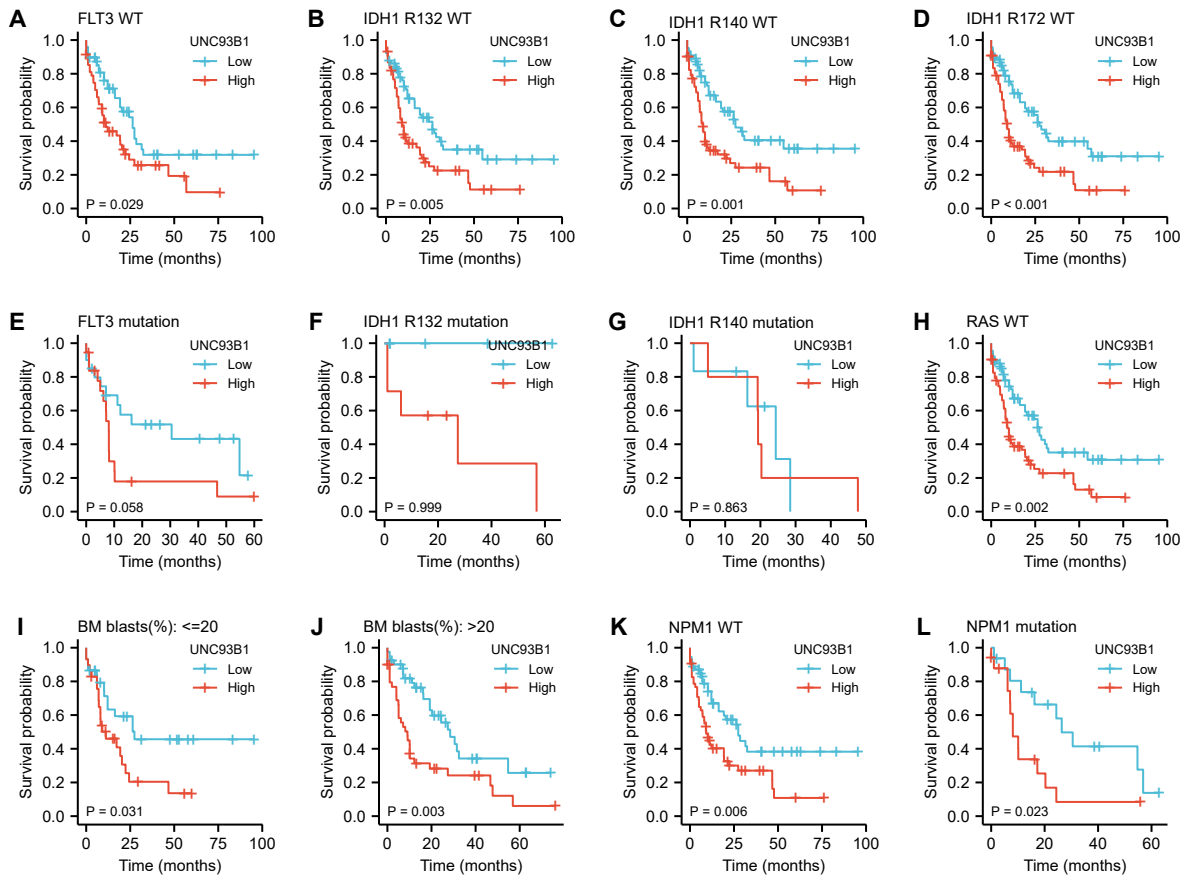

Supplement: Supplementary file 1 [file DataSheet2.PDF]

**A**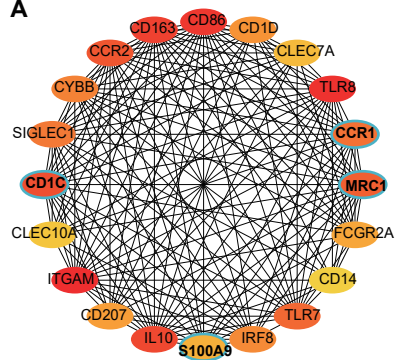**B**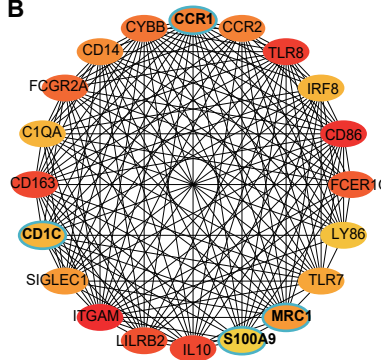**C**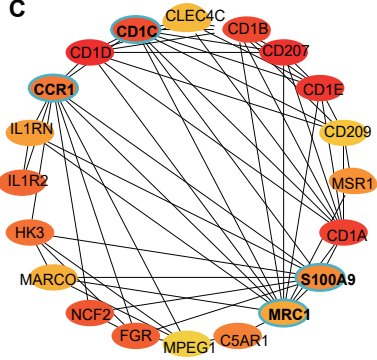

Supplement: Supplementary file 3 [file DataSheet4.PDF]

**A**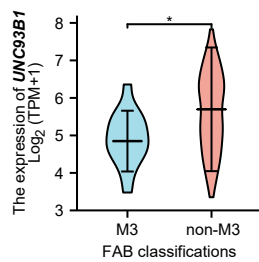**B**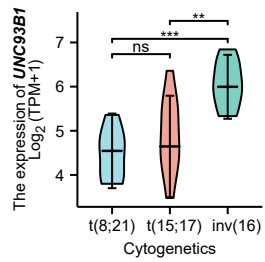

Supplement: Supplementary file 7 [file DataSheet1.PDF]
