# Supplementary material for: High-expression of the innate-immune related gene UNC93B1 predicts inferior outcomes in acute myeloid leukemia
Source: Front Genet. 2023 Jan 18;14:1063227. doi: 10.3389/fgene.2023.1063227 (PMC9891309; doi:10.3389/fgene.2023.1063227)
Supplement: Supplementary file 5 [file DataSheet3.PDF]

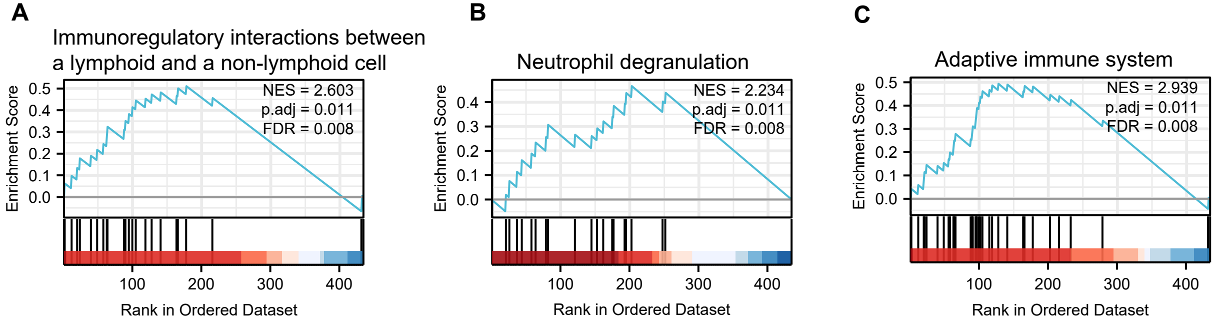

A
Immunoregulatory interactions between a lymphoid and a non-lymphoid cell
NES = 2.603
p.adj = 0.011
FDR = 0.008
Enrichment Score
0.5
0.4
0.3
0.2
0.1
0.0
100
200
300
400
Rank in Ordered Dataset
B
Neutrophil degranulation
NES = 2.234
p.adj = 0.011
FDR = 0.008
Enrichment Score
0.4
0.3
0.2
0.1
0.0
100
200
300
400
Rank in Ordered Dataset
C
Adaptive immune system
NES = 2.939
p.adj = 0.011
FDR = 0.008
Enrichment Score
0.5
0.4
0.3
0.2
0.1
0.0
100
200
300
400
Rank in Ordered Dataset
